# F175. NEUROLOGICAL SOFT SIGNS (NSS) AND BRAIN MORPHOLOGY IN PATIENTS WITH CHRONIC SCHIZOPHRENIA AND HEALTHY CONTROLS

**DOI:** 10.1093/schbul/sby017.706

**Published:** 2018-04-01

**Authors:** Christina Herold, Marc Lässer, Ulrich Seidl, Dusan Hirjak, Philipp Thomann, Marco Essig, Johannes Schröder

**Affiliations:** 1 University of Heidelberg; 2 Rehabilitation Hospital Zihlschlacht; 3 Center for Mental Health; 4 Central Institute of Mental Health, Medical Faculty Mannheim; 5 German Cancer Research Center

## Abstract

**Background:**

Subtle abnormalities in sensory integration, motor coordination and sequencing of complex motor acts or neurological soft signs (NSS) are a characteristic phenomenon in patients with schizophrenia at any stage of the illness. Previous MRI studies in schizophrenia have shown that NSS are associated with abnormal cortical, thalamic and cerebellar structure. However, these studies mainly focused on first-episode or recent onset schizophrenia and the respective correlates between brain structure and NSS in patients with a chronic course of the disorder remain rather unclear.

**Methods:**

49 middle-aged patients with chronic schizophrenia with a mean duration of illness of 20.3 ± 14.0 years and 29 healthy subjects matched for age and sex were included. NSS were examined on the Heidelberg Scale and correlated to grey matter by using whole brain high resolution magnetic resonance imaging (3 Tesla) with SPM12 analyses.

**Results:**

As expected, NSS in patients were significantly elevated in contrast to healthy controls, a finding, which not only applied to NSS sumscore, but also to the respective subscores motor coordination, sensory integration, complex motor tasks, right/left and spatial orientation and hard signs (p≤0.001).

Patients and healthy controls differed referring to right inferior frontal gyrus and left parahippocampal gyrus, with patients showing significantly reduced gray matter volumes, respectively. Within the patient group NSS total score was significantly correlated to reduced grey matter in right occipital lobe, left parahippocampal gyrus, left superior temporal gyrus, left thalamus (medial dorsal nucleus) and left posterior lobe of the cerebellum (declive). The respective findings remained significant after FDR correction for multiple comparisons (k=100 voxels). These results were confirmed when chlorpromazine (CPZ)-equivalents were introduced as additional covariate; moreover, no significant correlates arose between NSS and CPZ-equivalents. In the control group, VBM revealed that higher NSS total scores were significantly correlated with volume of right lentiform nucleus (medial globus pallidus).

**Discussion:**

Our study leads further support to the model of ‘cognitive dysmetria’ with a disrupted cortico-cerebellar-thalamic-cortical circuit in schizophrenia. This interpretation is also maintained by a different correlational pattern in our control group. Furthermore, the middle temporal/parahippocampal region may correspond to reduced mnestic functions, which are – besides elevated NSS – consistently reported to be impaired in patients with a chronic course of the disorder.

